# Demographic Characteristics, Nicotine Dependence, and Motivation to Quit as Possible Determinants of Smoking Behaviors and Acceptability of Shocking Warnings in Italy

**DOI:** 10.1155/2014/723035

**Published:** 2014-05-12

**Authors:** Alice Mannocci, Vittoria Colamesta, Vittoria Conti, Maria Sofia Cattaruzza, Gregorino Paone, Maria Cafolla, Rosella Saulle, Vincenzo Bulzomì, Daniele Antici, Pasquale Cuccurullo, Antonio Boccia, Giuseppe La Torre, Claudio Terzano

**Affiliations:** ^1^Department Public Health and Infection Disease, Sapienza University of Rome, Piazzale Aldo Moro 5, 00185, Rome, Italy; ^2^Respiratory Diseases Unit and ALS Respiratory and Critical Care Unit, Policlinico Umberto I-Sapienza University of Rome, 00185 Rome, Italy; ^3^Department of Cardiovascular, Respiratory, Nephrologic and Geriatric Sciences, San Camillo-Forlanini Hospital, Sapienza University of Rome, 00149 Rome, Italy; ^4^Gynaecology Obstetric Department, Casilino Hospital, 00169 Rome, Italy; ^5^Clinic Center, 80126 Naples, Italy; ^6^Fondazione Eleonora Lorillard Spencer Cenci, Sapienza University of Rome, 00185 Rome, Italy

## Abstract

*Introduction*. This paper presents the final results of a cross-sectional study started in 2010. It compares the perceived efficacy of different types of tobacco health warning (texts versus shocking pictures) to quit or reduce tobacco use. *Methods*. The study conducted between 2010 and 2012 in Italy enrolled adults smokers. Administering a questionnaire demographic data, smokers behaviors were collected. Showing text and graphic warnings (the corpse of a smoker, diseased lungs, etc.) the most perceived efficacy to reduce tobacco consumption or to encourage was quit. *Results*. 666 subjects were interviewed; 6% of responders referred that they stopped smoking at least one month due to the textual warnings. The 81% of the smokers perceived that the warnings with shocking pictures are more effective in reducing/quitting tobacco consumption than text-only warnings. The younger group (<45 years), who are more motivated to quit (Mondor's score ≥ 12), and females showed a higher effectiveness of shocking warnings to reduce tobacco consumption of, 76%, 78%, and 43%, respectively with *P* < 0.05. *Conclusions*. This study suggests that pictorial warnings on cigarette packages are more likely to be noticed and rated as effective by Italian smokers. Female and younger smokers appear to be more involved by shock images. The jarring warnings also appear to be supporting those who want to quit smoking. This type of supportive information in Italy may become increasingly important for helping smokers to change their behavior.

## 1. Introduction

Worldwide tobacco use continues to cause more deaths, nearly 6 million, and costs hundreds of billions of dollars of economic damage each year. If current trends continue, it will cause more than 8 million deaths annually by 2030 [1].

This high burden of tobacco is related to the causal association between smoking and a wide range of diseases. In fact, 10 types of cancer and 18 other diseases (including abdominal aortic aneurysm, acute myeloid leukemia, cataract, cervical cancer, kidney cancer, pancreatic cancer, pneumonia, periodontitis, and stomach cancer) are identified as smoking-related diseases [2, 3].

There has been a progressive decrease of the incidence of Italian smokers; in 50 years, the prevalence was estimated at 35.4%; recently, in 2012, the prevalence has fallen to 20.8%. (24.6% men and 17.2% women) [4]. In particular, over the past 5 years, the prevalence of smoking appears to remain fairly constant and fluctuates between 21% and 23%. This could be also due to the fact that the policies on smoking in Italy have not been very incisive as in the past. No coincidence that the last major legislation on the fight against smoking dates back to 2003 with the ban on smoking in public places. Also regarding the introduction of text warnings, which was in 1991 in Italy, this may not be the most effective, as these now may no longer be noticed as at the beginning. In the report, DOXA 2012, also, reports that the choice to consume a particular brand of cigarettes can also be influenced by the look of the package (colors, lettering, and graphics) with 1.5% a lot, 10.4% sufficiently, 32.8% not a lot, and 55% in no way [4]. Today the Italian population could be addicted to a type of warnings, so as not to feel the contents of the messages. This could lead to a difficulty in further reduction of the incidence of number of smokers. Health warnings on smoked tobacco products represent a significant area of tobacco control policy. Health warnings on tobacco packages are an important medium for communicating the tobacco-related risks to consumers.

The impact of health warning varies as “the pictorial warnings are more likely to be noticed, are associated with greater levels of engagement and emotional arousal, and may “wear out” more slowly than text-only warnings” [5]; “health warning labels had a greater cognitive and behavioral impact than either the abstract imagery or the text-only” [6]; the warnings increased the motivation to quit and reduce the number of cigarettes [7].

In Canada, graphic warnings have significant effect on smoking prevalence and quit attempts, decreasing the odds of being a smoker and increasing the odds of making a quit attempt [8].

In USA, the Food and Drug Administration (FDA) proposed 36 graphic warning labels and planned to select 9 to use. In young adults, the proposed labels had greater effects than text-only warning on fear-related reactions, smoking motivation, and discouragement with greater effect on nonsmokers than smokers. Images with babies or children or suffering or dead people or diseased body parts had the greatest effect on discouragement from smoking [9].

Also health warning could have a protective effect on recent quitters (<1 year) to stay a quitter [10].

In Europe, a recent study provides evaluation of the effectiveness of the text health warnings among daily cigarette smokers in four member states. The impact varies across the countries; in particular, it is higher where there is more comprehensive tobacco control programs among smokers with lowest socioeconomic status, who had made a quit attempt in the past year and smokers who smoked fewer cigarettes per day. The highest scores of Labels Impact Index (LII), used to quantify the effect, were found in France; while lower ones were in UK, the lowest scores were observed in Germany and The Netherlands [1].

This study represents the conclusion of a pilot survey published in 2012 [11]. In the previous paper, 243 current smokers were involved.

The main aim of the present study was to propose the final results expanding the sample size (*N* = 666) in order to verify the perception of the warning on cigarette packages in Italy in quitting smoking or in reducing the number of cigarettes smoked daily stratified by demographic characteristics, nicotine dependence, and motivation to quit. In addition, another objective of the study was to carry out a comparison of effectiveness between the text warnings, according to Italian law, compared to health warning graphics, according to some legislation in other countries.

In the present paper, the perceived efficacy to reduce tobacco consumption or to encourage quit was indicated using the following acronym: PERTC.

## 2. Methods

A cross-sectional study was conducted between June 2010 and September 2012 in three sites of Center and Southern Italy, Rome, Taranto, and Naples. The subjects enrolled were adult smokers (years ≥ 18).

The individuals were invited to complete a self-questionnaire in the waiting rooms of respiratory, orthopedic, or gynecological outpatient clinics, in the waiting rooms of a smoking cessation center, or during the hygiene lessons in the first year of the health professions students.

The STROBE statement was applied to present the results' study [12].

A multiple-choice questionnaire was developed to investigate the sociodemographic characteristics (age, gender) and smoke habits (number of cigarettes smoked daily, years of smoke) and how much actually the labels of the cigarettes packaging were appreciated and their perceived effectiveness for smoking cessation or reduction (see Table 1).

**Figure 1:**
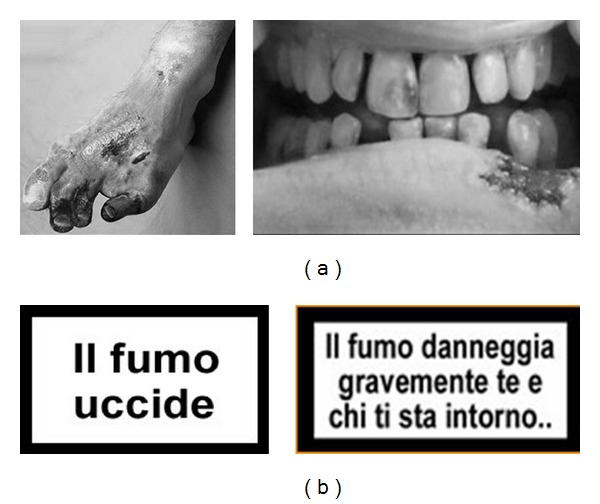
(a) Examples of pictorial health warnings. (b) An example of two textual warnings.

Addictional two different types of warnings were shown to the smokers during the interview (see Figures 1(a) and 1(b)): only text (current warning used in Italy, i.e., “Smoking kills”) and pictorial “shocking” warnings (i.e., the corpse of a smoker, diseased lungs, throat cancer, and rotting teeth). After that, to quantify the effect of the warning, two questions were asked: “If your favorite cigarettes brand decides to change his look using these pictorial warnings on tobacco packaging, would you think of buy another cigarettes brand?” Yes/no; “If you could choose the types of warning labels on cigarette packs, which one do you feel as more effective in helping to stop smoking?” Graphic images/texts/a combination of both.

In addition, the Fagerström and Mondor questionnaires [13, 14] were administered to estimate the nicotine dependence and motivation to stop smoking. The nicotine dependence and motivation to stop smoking were dichotomized (high versus low score) using as cutoff point Fagerström's score over 4 and Mondor's score ≥ 12, respectively.

### 2.1. Statistical Analysis

Statistical analysis was performed using SPSS for Windows (Statistical Package for the Social Sciences, Version 19; SPSS Inc., Chicago, IL, USA). Categorical data were shown as absolute frequencies and percentages. Continuous data were presented as means ± standard deviation (SD) or medians (interquartile ranges, IR), as appropriate.

The nicotine dependence and motivation to stop smoking and the associations by gender and age groups were compared by the chi-square test or the Fisher's exact test whenever the sample sizes were rather small.

The following two logistic multivariate regression models were computed using as outcome two questions concerning the impact of the graphic warnings:

- if shocking images were used on cigarette boxes, would they have greater effect than simple warning text currently used? Yes/no;
- if your favorite cigarettes brand decides to change his look using pictorial warnings on tobacco packaging, would you think of buying another cigarette brand? Yes/no.

The independent factors included in the models were followed dummies variables: gender, age groups (<45 years), nicotine dependence (high/low), and motivation to stop smoking (high/low). The ORs adjusted for the covariates with CI95% were indicated.

The Hosmer and Lemeshow test was applied to estimate the goodness of fit for each model.

The statistical significance was set at *P* < 0.05.

## 3. Results

A total of 666 smokers entered the study; 47.5% (*N* = 313) of the responders was male; 49.9% (*N* = 332) had <45 years old (mean  age = 45 years; SD = 17.5 years).

The mean duration of tobacco use was 24 years (SD = 16.6 years) (data are not reported in table); 58.9% of the sample smoked 10–25 cigarettes per day, 27.8% less than 10, and 13.3% more than 25.

68.5% of smokers had medium high nicotine dependence (Fagerström's score > 4) and 51.4% a low motivation to quit (Mondor's score < 12); it has not been possible to calculate the scores in 9.5% of the cases due to missing values.

Table 1 shows the answers obtained. Almost all of the subjects (98%) were aware of health consequences. Concerning the short-term effects of tobacco consumption, the major worries referred were breathlessness (50.5%), yellow teeth (20%), halitosis, or bad breath (20.3%).

A very low percentage of smokers referred that they stopped smoking at least one month due to the warnings (6.2%), so also there is a soft influence on the smokers habits, in particular 12.5%, that referred a reduction of the daily number of cigarettes smoked, and 8.2% do not smoke just waking up in the morning or after drinking a cup of coffee.

One fourth of the sample (24.3%) did not know that individuals smoking* light*,* mild*, and* blue* cigarettes are likely to inhale the same amount of hazardous chemicals and remain at high risk for developing smoking-related cancers and other diseases.

62% of the smokers declared that the warnings with shocking pictures have a more effective communication in order to reduce/quit tobacco consumption than text-only warnings, and the combinations of text and shocking images were the most preferred (46.6%). In addition one smoker out of four (23.7%) would have changed the brand of cigarettes if it decided to print shocking pictorial advertisements on smoking health damages.

Table 2 shows the comparison by gender and age groups. Women seemed to be more sensitive by the effects of smoking on the physical aspect, in particular, on wrinkles and smelly clothes (22% *P* < 0.001; 17% *P* = 0.020); they seem to have been impressed by the current warnings reducing the daily consumption of tobacco (15% of women versus 9% of men, *P* = 0.020), and they would be inclined not to buy their favorite package if there are printed gruesome images on the health effects of smoking (43% female versus 32% male, *P* = 0.025).

The actual textual warning had a different effect on persuading to not inhale tobacco: 87% in women versus 94% in men (*P* = 0.002).

On the other hand, the males were more worried about the physical fitness with special reference to the breathlessness (56% versus 45%, *P* = 0.003); they had stopped smoking for a short time thanks to the warnings (8% versus 4%, *P* = 0.034) even though they then restarted smoking again as before (75%).

Young smokers (<45 years) showed a significantly higher worry for those aspects related to both the appearance of the face and the physical form than the older ones: wrinkles, skin spots, halitosis, yellow teeth and nails, and hair loss.

Younger age group also showed a greater attention to warnings, curiosity and bad knowledge about tobacco, and the damage it caused (93% versus 88%, *P* = 0.034; 89% versus 82%, *P* = 0.009; 42% versus 30%, *P* < 0.001; 20% versus 29%, *P* = 0.022) and they were also more sensitive to pictorial warnings (85% versus 76%, *P* = 0.019).

The comparison of the two different dependences on smoking and motivation to quit groups shows some significant differences (Table 3).

The group more motivated to stop smoking, in comparison with the one not motivated, is more informed (resp., 89% versus 82%, *P* = 0.019) and considers the labels crucial to increase the awareness and the motivation to reduce/quit tobacco consumption (*P* = 0.021), and it was not by chance that this group considers the warnings with graphic text most appropriate to fight the habit of smoking (*P* = 0.003). The higher significant percentage of motivated smokers reported that the health warning increase the curiosity (42% versus 31%, *P* = 0.023). The consequences more referred in the high motivated groups are breathlessness, skin spots, and yellow fingernails.

In the group that shows a high nicotine dependence, there was bigger unawareness of the effects of smoking on health (78% versus 69%, *P* = 0.044) and a less impressionability by shocking images (78% versus 87%, *P* = 0.018).

The multivariate logistics analysis confirmed significant higher effect the shocking warnings in younger and lower dependent smokers (see Table 4), respectively, OR = 0.59 for older smokers (CI95%: 0.36–0.97) and OR = 0.46 for high nicotine dependence (CI95%: 0.26–0.84). No significant difference was found concerning changing the favorite cigarette brand in case the company adopts the shock labeling.

## 4. Discussion

According to the TNS Qualitative Eurobarometer study [15], there is a perception that people have become desensitized to health warnings labels and that they now “blend into” the packaging. Although most respondents claimed that they would not be motivated by health warnings alone to give up smoking, they felt they were important and should continue to feature on tobacco packaging.

Moreover, in agreement with several studies [8, 16–20], the present findings showed that higher motivated to quit respondents who had been exposed to pictorial health warnings expressed the view that these were more impactful than text-only warnings (89% versus 78%, *P* = 0.003). Consequently, it is possible to conclude that pictorial warnings should be more widely adopted instead of or in combination with text to help those who are already inclined to quit.

The typology of shocking warnings does not appear to be noticed in those who exhibit different levels of nicotine dependence. The Fagerström score does not highlight differences in the choice of the best type of warning to discourage tobacco consumption.

The pilot study [11] and the evidence suggest that pictorial warning labels, particularly those with graphic images, may be more persuasive among female populations [21, 22]; in the present study, 43% of the female respondents talked about changing the packet to avoid seeing the “shocking” image in comparison with the 32% of male ones.

According to the previous study, this research shows that the respondents aged <45 years were more sensitive to warnings (54% present study; 65% previous study) [11]. The association with younger people is also evident in several studies too [21, 23, 24].

About a third of respondents (36.2%) said that the labels have increased curiosity about tobacco or desire to be better informed or to be helped to give up smoking, although in the lower dependence and higher motivated to quit groups the interest is around 40%. Some studies suggest including telephone number/more information on clinics/pharmacies/a website address on the packets [25, 26] or including a reason why people should seek help, to improve the credibility of the message.

The study has a number of limitations. It is based on an opportunistic sample of smokers who voluntarily decided to take part in the study. In addition, the sample included different settings (general population but also respiratory disease patients and participants of smoking cessation programs). This may have introduced a selection bias in the results and, in fact, may have involved only special categories of smokers, for example, the most sensitive to the health problems group or hardcore group of smokers. To control some of these limits, the multivariate logistic model was applied using age, dependence to smoking, and motivation to quit.

It was also not counted the number of individuals who refused to fill in the questionnaire, and even the reasons for the rejection: maybe the most important limitation is not having evaluated if the questionnaire is usable for all types of smokers. This consideration could explain the high number of missing answers regarding the impression on the shocking images (between 18% and 39%) in which it is not possible to give a correct interpretation: have the pictures disturbed and ill-disposed responders?

The impacts of the introduction of graphic health warnings on cigarette packs on the smoking-related behaviors, perceptions, and intentions of adolescents were not investigated.

The work looked cross-sectionally at the association between cognitive processing of the warning labels and smoking habits. The longitudinal analyses would have been conducted to determine if the graphic warning labels altered smoking intentions and behaviors.

In addition, the information collected in this study is self-reported and therefore can be affected by many factors. For example, the impact of a message or the its understanding can be influenced by social, demographic, environmental, and political characteristics and the responder's sensitivity may depend on the presence or absence of certain personal conditions.

This research suggests that, beyond communicating the health risks of smoking and protecting nonsmokers from the harmful effects of environmental tobacco smoke, the pictorial shocking warnings encourage to stop smoking or “to horrify” for good purposes the ones more motivated to quit, the young, and the female smokers. Despite these very encouraging results in some countries, the adoption of this way of communicating the health risks has not yet been adopted. Most likely, the introduction of warnings of this type is mainly slowed by economic interests of the tobacco industry [27].

Although tobacco companies have suggested that pictorial warnings “annoy” smokers, this research in accordance with the literature suggests that, overall, smokers welcome more health information on their packages, including information that presents the health consequences of smoking in a vivid, arousing manner [28].

In conclusion, the findings in accordance with the previous publication show that female and younger smokers appear to be more involved by shocking images. The jarring warnings appear to be supporting those who want to quit smoking. This is an Italian study that sought to strengthen the evidence already known in the international literature but which had not yet been explored in this country and provide additional reasons to the health advocates and policy bodies to pursue more efficient smoke-free and tobacco labeling policies.

## Figures and Tables

**Table 1:** Description of the sample.

Variables		*N*	(%)	Missing*
Gender	Female	346	52.5	7
	Male	313	47.5	

Age	<45 years	332	49.9	1
	≥45 years	333	50.1	

Number of daily cigarettes	0–9	184	27.8	4
	10–25	390	58.9	
	>25	88	13.3	

Years of smoking	<6 years	97	14.6	2
	6–15 years	170	25.6	
	16–25 years	120	18.1	
	>25 years	277	41.7	

Fagerström score (nicotine dependence)	High	413	68.5	63
	Low	190	31.5	

Mondor's score (motivation to quit)	High	293	48.6	63
	Low	310	51.4	

Are you aware of the damage caused by smoking?	Yes	643	98	10
	No	13	2	

What is the consequence that in the immediate worries you? (Multiple answer question)	Wrinkles	104	15.6	1
	Skin spots	104	15.6	
	Breathlessness	336	50.5	
	Halitosis or bad breath	135	20.3	
	Yellow teeth	133	20	
	Yellow fingernails	10	2.6	
	Hair loss	13	3.1	
	Bad smelling clothes	92	13.8	

Have you ever stopped smoking due to the warnings?	Yes	41	6.2	9
	No	616	93.8	

Are you or have you been influenced by the health warnings on cigarettes packages (in relation to the daily number of cigarettes smoked)?	Yes No	80 560	12.5 87.5	26

Have you changed your smoking habits due to the warnings (e.g., do not smoke after coffee)?	Yes	52	8.2	28
	No	586	91.8	

If yes. Have you returned to your previous smoking habits after a short time?°	Yes	25	69.4	14
	No	11	30.6	

Having read the smoking warnings on packages. Do you inhale it?	Yes	596	90.3	6
	No	64	9.7	

Do you consider it important to report the health warnings about tobacco consumption on cigarette packs?	A lot	170	25.9	9
	Enough	176	26.8	
	Poor	186	28.3	
	No	125	19.0	

Have the messages communicated that smoking ruins your health?	Yes	563	87.6	23
	No	80	12.4	

Have the messages communicated to you that smoking causes damage to those around you, such as your children or family members?	Yes No	534 92	85.3 14.7	40

Have the health warnings increased the curiosity or the desire to be better informed or to be helped to give up smoking?	A lot	50	7.6	9
	Enough	188	28.6	
	Poor	258	39.3	
	No	161	24.5	

Do you think that the light, blue, gold, and mild cigarettes are less hazardous than regular cigarettes?	Yes	140	24.3	91
	No	435	75.7	

If shocking images were used on cigarette boxes, would they have greater effect than simple warning text currently used?	Yes	395	62.0	29
	No	95	14.9	
	Do not know	147	23.1	

If you could choose the types of warning labels on cigarette packs, which one do you feel as more effective in helping to stop smoking?	Textual health warning	92	14.2	16
	Graphic health warnings with shocking images	152	23.4	
	Both shocking images with text	303	46.6	
	Do not know	103	15.8	

If your favorite cigarettes brand decided to change the look of its cigarette boxes with shocking images on smoking health damages, would you think of changing it?	Yes	154	23.7	16
	No	250	38.5	
	Do not know	246	37.8	

*Missing values or answer “I do not know.”

°The sample size in this case is 36.

**Table 2:** Description and comparison by gender and age groups. For dichotomous variables (yes/no), “Yes” percentage was reported only.

Variables		Male *N* (%)	Female *N* (%)	*P* ^∧^	<45 years *N* (%)	≥45 years *N* (%)	*P* ^∧^
Are you aware of the damage caused by smoking?	Yes	309 (99)	329 (97)	0.070	323 (98)	320 (99)	0.420

What is/are the consequence/s that in the immediate worries you? (Multiple-answers)	Wrinkles	27 (9)	77 (22)	**<0.001**	78 (24)	26 (8)	**<0.001**
	Skin spots	42 (40)	62 (60)	0.110	67 (20)	37 (11)	**0.001**
	Breathlessness	176 (56)	154 (45)	**0.003**	162 (49)	173 (52)	0.393
	Halitosis or bad breath	58 (19)	76 (22)	0.266	94 (28)	40 (12)	**<0.001**
	Yellow teeth	56 (18)	77 (22)	0.158	97 (29)	36 (11)	**<0.001**
	Yellow fingernails	4 (2)	7 (3)	0.445	11 (7)	0 (0)	<0.001*
	Hair loss	3 (2)	10 (5)	0.071	10 (6)	3 (1)	**0.004**
	Bad smelling clothes	33 (11)	58 (17)	**0.020**	50 (15)	41 (12)	0.310

Have you ever stopped smoking due to the warnings? (Decision to quit)	Yes	25 (8)	14 (4)	**0.034**	15 (5)	26 (8)	0.067

Are you or have you been influenced by the health warnings on cigarettes packages (in relation to the daily number of cigarettes smoked)? (Foregoing of cigarettes)	Yes	28 (9)	51 (15)	**0.020**	37 (11)	43 (14)	0.370

Have you changed your smoking habits due to the warnings (e.g., do not smoke after coffee)? (Forgoing of cigarettes)	Yes	23 (7)	29 (9)	0.583	29 (9)	23 (7)	0.497

°If yes. Have you returned to yours previous smoking habits after a short time?	Yes	12 (75)	13 (65)	0.718*	13 (77)	12 (63)	0.387

Having read the smoking warnings on packages, do you inhale it?	Yes	294 (94)	297 (87)	**0.002**	306 (93)	289 (88)	**0.034**

Do you consider it important to report the health warnings about tobacco consumption on cigarette packs?	A lot	83 (27)	84 (25)	0.468	98 (30)	71 (22)	**0.048**
	Enough	82 (27)	92 (21)		92 (28)	84 (26)	
	Poor	94 (30)	92 (27)		82 (25)	104 (32)	
	No	52 (17)	72 (21)		58 (18)	67 (21)	

Have the messages communicated that smoking ruins your health? (Thoughts of harm)	Yes	268 (88)	290 (87)	0.929	293 (90)	270 (85)	0.071

Have the messages communicated to you that smoking causes damages to those around you, such as your children or family members? (Thoughts of harm)	Yes	252 (85)	280 (86)	0.641	287 (89)	246 (82)	**0.009**

Have the health warnings increased the curiosity or the desire to be better informed or to be helped to give up smoking? (Warning salience)	A lot	27 (9)	21 (6)	0.153	21 (6)	29 (9)	**<0.001**
	Enough	83 (27)	104 (31)		120 (36)	68 (21)	
	Poor	132 (42)	124 (36)		116 (35)	142 (44)	
	No	69 (22)	92 (27)		74 (22)	87 (27)	

Do you think that the light, blue, gold, and mild cigarettes are less hazardous than regular cigarettes?	Yes	67 (25)	73 (24)	0.762	58 (20)	82 (29)	**0.022**

If shocking images were used on cigarette boxes, would they have greater effect than simple warning text currently used?	Yes	194 (83)	197 (78)	0.189	215 (85)	180 (76)	**0.019**

If your favorite cigarettes brand decide to change the look of its cigarette boxes with shocking images on smoking health damages, would you think of changing it?	Yes	60 (32)	92 (43)	**0.025**	81 (40)	73 (36)	0.390

If you could choose the types of warning labels on cigarette packs, which one do you feel as more effective in helping to stop smoking?	Textual health warning	45 (17)	46 (17)	0.985	43 (15)	49 (19)	0.480
	Graphic health warnings with shocking images	72 (27)	77 (28)		81 (28)	71 (27)	
	Both shocking images with text	148 (56)	155 (56)		163 (57)	140 (54)	

^∧^ *P* value chi-square test; *P value Fisher's exact test; °the sample size in this case is 36; bold: *P* < 0.05.

**Table 3:** Description and comparison by motivation to quit and smoke dependence groups. For dichotomous variables (yes/no), “yes” percentage was reported only.

Variables		Low motivation *N* (%)	High motivation *N* (%)	*P* ^∧^	Low dependence *N* (%)	High dependence *N* (%)	*P* ^∧^
Are you aware of the damage caused by smoking?	Yes	299 (98)	284 (98)	0.629	182 (97)	403 (98)	0.208*

What is/are the consequence/s that in the immediate worries you? (Multiple answers)	Wrinkles	46 (15)	55 (19)	0.196	29 (15)	70 (17)	0.604
	Skin spots	64 (21)	39 (13)	**0.017**	31 (16)	69 (17)	0.904
	Breathlessness	141 (46)	161 (55)	**0.020**	92 (48)	212 (51)	0.507
	Halitosis or bad breath	65 (21)	64 (22)	0.793	37 (20)	86 (21)	0.702
	Yellow teeth	56 (18)	70 (24)	0.079	38 (20)	86 (21)	0.816
	Yellow fingernails	9 (5)	2 (1)	**0.024**	5 (4)	5 (2)	0.302*
	Hair loss	4 (2)	8 (4)	0.290	2 (2)	9 (4)	0.514*
	Bad smelling clothes	51 (17)	38 (13)	0.228	21 (11)	60 (15)	0.245

Have you ever stopped smoking due to the warnings? (decision to quit)	Yes	18 (6)	18 (6)	0.885	13 (7)	26 (6)	0.817

Are you or have you been influenced by the health warnings on cigarettes packages (in relation to the daily number of cigarettes smoked)? (Foregoing of cigarettes)	Yes	31 (10)	43 (15)	0.090	28 (15)	44 (11)	0.175

Have you changed your smoking habits due to the warnings (e.g., do not smoking after coffee)? (Foregoing of cigarettes)	Yes	22 (7)	25 (9)	0.515	17 (9)	29 (7)	0.444

°If yes. Have you returned to your previous smoking habits after a short time?	Yes	11 (79)	12 (67)	0.457*	9 (56)	14 (82)	0.141*

Having read the smoking warnings on packages, do you inhale it?	Yes	280 (91)	265 (90)	0.943	162 (85)	386 (94)	0.001

Do you consider it important to report the health warnings about tobacco consumption on cigarette packs?	A lot	63 (21)	90 (31)	**0.021**	52 (27.5)	97 (24)	0.087
	Enough	95 (31)	68 (23)		52 (27.5)	116 (28)	
	Poor	89 (29)	83 (28)		60 (32)	110 (27)	
	No	60 (19)	52 (18)		25 (13)	88 (21)	

Have the messages communicated that smoking ruins your health? (Thoughts of harm)	Yes	258 (86)	259 (90)	0.150	169 (90)	347 (87)	0.249

Have the messages communicated to you that smoking causes damage to those around you, such as your children or family members? (Thoughts of harm)	Yes	245 (82)	247 (89)	**0.019**	160 (87)	328 (84)	0.296

Have the health warnings increased the curiosity or the desire to be better informed or to be helped to give up smoking? (Warning salience)	A lot	18 (6)	24 (8)	**0.023**	17 (9)	27 (7)	0.070
	Enough	78 (25)	100 (34)		60 (32)	115 (28)	
	Poor	125 (41)	111 (38)		80 (42)	158 (38)	
	No	86 (28)	58 (20)		33 (17)	111 (27)	

Do you think that the light, blue, gold, and mild cigarettes are less hazardous than regular cigarettes?	Yes	72 (26)	57 (22)	0.305	52 (31)	79 (22)	**0.044**

If shocking images were used on cigarette boxes, would they have greater effect than simple warning text currently used?	Yes	178 (78)	183 (84)	0.114	124 (87)	236 (78)	**0.018**

If your favorite cigarettes brand/company decide to change the look of its cigarette boxes with shocking images on smoking health damage, would you think of changing it?	Yes	71 (37)	73 (41)	0.424	53 (46)	93 (37)	0.095

If you could choose the types of warning labels on cigarette packs, which one do you feel as more effective in helping to stop smoking?	Textual health warning	58 (22)	27 (11)	**0.003**	22 (14)	62 (18)	0.319
	Graphic health warnings	65 (25)	71 (29)		41 (26)	94 (28)	
	Both shocking images with text	137 (53)	149 (60)		97 (61)	184 (54)	

^∧^ *P* value chi-square test; *P value Fisher's exact test; °the sample size in these cases is 32; bold: *P* < 0.05.

**Table 4:** Multivariate logistic models to evaluate the effect of the pictorial shocking warnings.

Independents variables	Outcomes			
	Agree with the fact that the shocking images used on cigarette boxes have greater effect than simple warning text currently used		Agree to change your favorite cigarettes brand if a new look with shocking images on smoking health damage was adopted by the cigarette company	
	OR	CI 95%	OR	CI 95%
<45* versus ≥45 years	**0.59**	**0.36–0.97**	0.96	0.61–1.50
Male* versus female	0.64	0.39–1.07	0.68	0.44–1.07
Low* versus high motivation to quit	1.30	0.78–2.15	0.62	0.58–1.39
Low* versus high nicotine dependence	**0.46**	**0.26–0.84**	1.5	0.94–2.38

Hosmer and Lemeshow's *P* value test			0.46	

*Reference group.
